# Glycemic variability assessed by continuous glucose monitoring in hospitalized patients with community-acquired pneumonia

**DOI:** 10.1186/s12890-022-01874-7

**Published:** 2022-03-09

**Authors:** Mikkel Thor Olsen, Arnold Matovu Dungu, Carina Kirstine Klarskov, Andreas Kryger Jensen, Birgitte Lindegaard, Peter Lommer Kristensen

**Affiliations:** 1 Department of Endocrinology and Nephrology, Copenhagen University Hospital – North Zealand, Hilleroed, Denmark; 2 Department of Pulmonary- and Infectious Diseases, Copenhagen University Hospital – North Zealand, Hilleroed, Denmark; 3 Department of Clinical Medicine, Faculty of Health and Medical Sciences, Copenhagen University Hospital – North Zealand, Hilleroed, Denmark; 4 Biostatistics, Department of Public Health, University of Copenhagen, Øster Farimagsgade 5, 1014 Copenhagen, Denmark; 5 Department of Clinical Research, Copenhagen University Hospital – North Zealand, Hilleroed, Denmark

**Keywords:** Chronic obstructive pulmonary disease, Community-acquired pneumonia, Continuous glucose monitoring, Diabetes mellitus, Glucocorticoid-induced hyperglycemia, Glycemic variability, Length of stay

## Abstract

**Background:**

Glycemic variability (GV) has only been sparsely studied in patients with community-acquired pneumonia (CAP). This study aimed to quantify in-hospital GV in CAP patients, including determining the impact of type 2 diabetes mellitus (T2DM) and glucocorticoid (GC) treatment on GV.

**Methods:**

This is a prospective cohort study of CAP patients (N = 40) with or without T2DM and treated or not with GCs. The primary endpoint was GV measured as glucose standard deviation (SD), coefficient of variation (CV), and postprandial glucose excursions (PPGE) based on continuous glucose monitoring (CGM). Analysis of glucose data was split into daytime and nighttime when possible.

**Results:**

Patients included had a mean age of 74 (range 55 to 91) years. SD (95%CI) increased by a factor of 1.93 (1.40 to 2.66) and 2.29 (1.38 to 3.81) in patients with T2DM and not treated with GCs during the daytime and the nighttime, respectively (both *P* < 0.01), and by a factor of 1.42 (1.04 to 1.97) in patients treated with GCs but without T2DM during the daytime (*P* = 0.031) compared to patients without T2DM and not treated with GCs. CV (95%CI) increased by 5.1 (0.0 to 10.1) and 8.1 (1.0 to 15.2) percentage points during the daytime and the nighttime, respectively, in patients with T2DM and not treated with GCs compared to patients without T2DM and not treated with GCs (*P* = 0.046 and *P* = 0.026, respectively). PPGE (95% CI) increased during lunch by 2.5 (0.7 to 4.3) mmol/L (45 (13 to 77) mg/dL) in patients with T2DM and treated with GCs compared to patients without T2DM and not treated with GCs (*P* = 0.018).

**Conclusions:**

CAP patients receiving GCs, especially those with T2DM, are at great risk of developing high GV and therefore require clinical attention to mitigate GV. This applies particularly during the daytime. Results support the 1 to 2-h post-lunch screening procedure for glucocorticoid-induced hyperglycemia in patients without diabetes. SD was positively correlated with hospital length of stay.

**Supplementary Information:**

The online version contains supplementary material available at 10.1186/s12890-022-01874-7.

## Introduction

Worldwide, community-acquired pneumonia (CAP) is a leading course of death and morbidity [1]. In Denmark, a 30-day mortality rate of 11% has been reported in hospitalized pneumonia patients [2] and up to 20% for patients with both pneumonia (all types, except coronavirus disease 2019 (COVID-19)) and diabetes mellitus type 2 (T2DM) [3]. Hospitalized CAP patients with hyperglycemia at admission have an increased risk of mortality and intensive care unit admission compared to patients with normoglycemia [4]. Glycemic dysregulation in hospitalized patients is multifactorial and related to the acute stress response (i.e., disease severity), diabetes status, and treatment modalities known to induce hyperglycemia, for example exogenous glucocorticoids (GCs) [5].

Chronic obstructive pulmonary disease (COPD) is common among CAP patients. Currently, treatment with systemic GCs is the standard of care for CAP patients with COPD in acute exacerbation and decreases the length of stay (LOS), time to clinical stability, and mortality [6]. However, GCs may cause glucocorticoid-induced hyperglycemia (GIH) and increased glycemic variability (GV) which could potentially counteract the beneficial effects of GCs. The explanation for the adverse effects of hyperglycemia in CAP patients such as increased mortality, increased LOS and other in-hospital complications could represent an effect of hyperglycemia per se [7], but GV could be involved too [8]. High short-term GV is associated with increased LOS and mortality in critically ill patients [9] and in non-critically ill patients independent of admission diagnosis [10]. High GV also increases superoxide production, oxidative stress, endothelial dysfunction, and inflammatory cytokine overproduction and increases the risk of hypoglycemia which increases inflammatory cytokine levels, platelet activation and endothelial dysfunction [8].

In the few published studies about GV in CAP patients, authors reported an association between high GV and increased LOS [11], while others did not [12]. However, blood glucose was measured only two to four times per day on average and not necessarily on consecutive days. A more precise quantification of GV using continuous glucose monitoring (CGM), which provides information about glucose trajectories with intervals of five minutes, may therefore prove to be a more adequate tool for assessment of GV than standard point-of-care (POC) blood glucose testing. As standard measurements of GV, an international CGM consensus report recommends registration and reporting of the variation around the mean blood glucose, i.e. standard deviation (SD) and SD relative to the mean glucose level as a percentage, i.e. coefficient of variation (CV) [13]. Postprandial glucose excursion (PPGE) is also a measurement of GV and is especially relevant in patients treated with GCs since GCs are assumed to cause postprandial hyperglycemia [14]. This study aimed to investigate to what extent GV, measured as SD, CV, and PPGE, is influenced by both treatment with GCs and a diagnosis of T2DM in patients hospitalized with CAP by use of CGM.

## Method and materials

### Study population

We identified CAP patients with or without T2DM and treated or not with GCs hospitalized at Copenhagen University Hospital – North Zealand, Denmark between February 2021 and June 2021.

We constructed four equal-sized groups of patients, all with CAP (N = 40): Group 1: CAP, group 2: CAP and GC treatment, group 3: CAP and T2DM, and group 4: CAP, GC treatment, and T2DM. Diabetes diagnosis was obtained through patients’ journals. To limit confounding, patients were matched by age and gender, and HbA1c was measured for all patients to avoid undiagnosed diabetes in groups 1 and 2. Acute exacerbation of COPD ended up being the only reason for GC treatment in this study. The GC regimen followed regional standard care with IV methylprednisolone (Solu-Medrol®) 40 mg followed by four days of oral prednisolone 37.5 mg per day.

### Inclusion and exclusion criteria

Patients who met the following criteria were eligible for inclusion: Age ≥ 18 years, radiologically verified pneumonia and at least one symptom and/or clinical signs of pneumonia (i.e. cough, chest pain, dyspnoea, temperature ≥ 38.0 °C or < 35.0 °C, pathological auscultation) and informed written consent.

The exclusion criteria were COVID-19, incapacitation, known hypersensitivity to the band-aid of the glucose sensor, parenteral nutrition, and pancreatic disorders.

### Data collection

Continuous glucose monitoring was performed using iPro2® as the recorder and an Enlite® glucose sensor (Medtronic, Northridge, CA). The CGM system was inserted in the abdominal area according to the manufacturer’s guidelines. Recordings by CGM were fully blinded during hospitalization and therefore not used for in-hospital diabetes management. Standard POC capillary blood glucose measurements were performed by ward glucometers FreeStyle Precision Pro® (Abbot, Berkshire, UK) three times daily before main meals (7:00 AM, 12:00 AM, and 5:00 PM) to calibrate the CGMs. CGM data were collected from study enrollment until discharge. At least 24 h of CGM data were required to be included in the analyses.

At baseline, we collected clinical data (age, gender, comorbidities, CURB-65 score as a measurement of severity of CAP, early warning score, Charlson comorbidity index, and medications before admission) and standard blood work (hemoglobin A1c (HbA1c)). For the two groups receiving GCs, CGM data were analyzed only during GC exposure which was defined as the period between the first GC dose and 24 h (six half-lives for prednisolone) after GCs were stopped. Meal registration (timing and percentage of the amount of meal consumed at breakfast, lunch, and dinner) made it possible to calculate PPGE.

### Primary outcomes

GV was evaluated as SD of all CGM-glucose values, CV of all CGM-glucose values, and PPGE. PPGE was defined as the difference between the blood glucose level before meal start and the highest blood glucose level within two hours after meal start [15]. SD and CV represent the overall amplitude of GV, while PPGE characterizes GV in relation to meals. CV is less influenced by fluctuations in mean glucose level and HbA1c compared to SD [16] but should otherwise be interpreted in the same way. Only meals with an intake of at least 25% of the plate were included in these calculations.

### Secondary outcomes

We assessed the percentage of time of CGM-glucose values spent in Time In Range (TIR) (3.9–10.0 mmol/L or 70–180 mg/dL), Time Above Range (TAR) (> 10.0 mmol/L or > 180 mg/dL), Time Below Range (TBR) (< 3.9 mmol/L or < 70 mg/dL) and mean glucose level (mmol/L and mg/dL). We also report the glycemic gap defined as the difference between the HbA1c-derived average glucose level [17] before admission and the mean glucose level during hospitalization (mmol/L).

### Statistical analysis

Data analyses were split into daytime from 07:00:00 AM to 10:59:59 PM and nighttime from 11:00:00 PM to 06:59:59 AM when relevant. Categorical variables were compared between the four groups with Pearson’s chi-squared test. ANOVA for normally distributed data and Kruskal–Wallis test for skewed data were applied to assess differences in continuous variables.

**Fig. 1:**
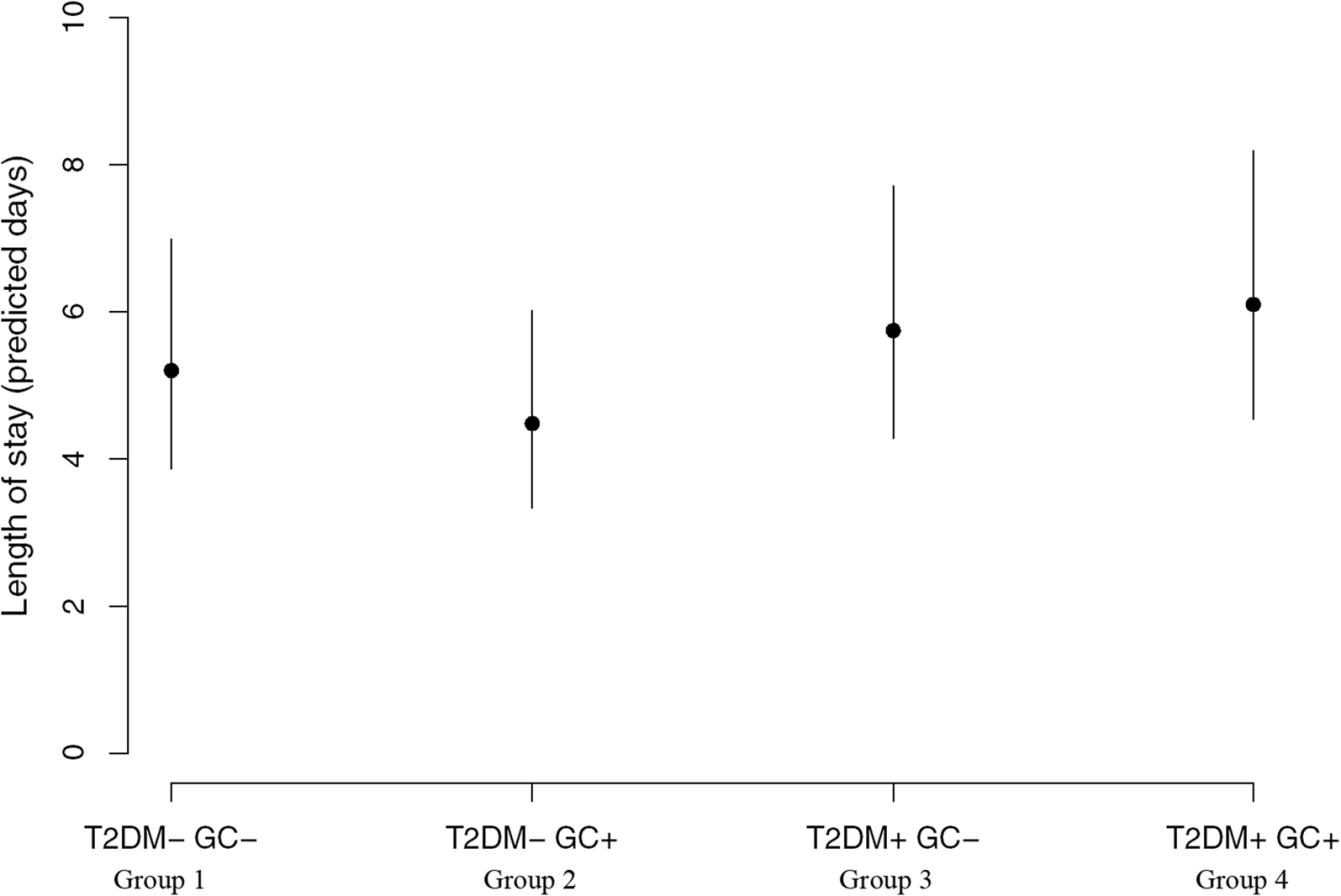
Prediction of length of stay at group levels. Dots depict the predicted (Model 1) length of stay for Groups 1 to 4. Lines depict the 95% CI

A linear regression analysis was done to assess the effect of T2DM and GCs (explanatory variables) and a possible interaction between T2DM and GCs on primary and secondary outcomes (dependent variables). The SD variable was logarithmically transformed. For repeated measurements (of PPGE, insulin dose, and food intake), a linear mixed model was used to determine the effect of T2DM and GCs on the outcome PPGE and for determining any statistical difference among groups regarding food intake and IE of insulin per day in Table 1. For the TAR and TBR outcomes, a zero adjusted Gamma distribution [18] was used to accommodate the positivity and exact zero in the outcomes. Post hoc analyses were performed for all outcomes, adding HbA1c, BMI, and CURB-65 as explanatory variables. To analyze an association between GV and LOS we did a post hoc linear regression analysis and included SD and CV of all CGM-glucose values during the daytime and the nighttime (Model 1 and Model 2, respectively), T2DM status (yes/no), GC status (yes/no) and Charlson comorbidity index as explanatory variables. LOS per patient was registered as days, hours, and minutes and was considered as a continuous variable. The LOS variable was logarithmically transformed. Figure 1 depicts the predicted (by Model 1) LOS for Groups 1 to 4. To make Fig. 1 by use of Model 1, we used the group characteristics as input to Model 1, i.e. T2DM status (yes/no) and GC status (yes/no), and for continuous variables, we used the means for Groups 1 to 4 as inputs.

**Table 1:** Demographic and clinical characteristics of 40 hospitalized patients with community-acquired pneumonia with or without diabetes, treated or not with glucocorticoids

	CAP	CAP + GC	CAP + T2DM	CAP + GC + T2DM	*P*-value
	n = 10	n = 10	n = 10	n = 10	
Age (years), mean (SD)	71.2 (11.7)	73.5 (7.7)	74.9 (10.9)	74.2 (9.5)	0.886
median (range)	71.5 (55.0 to 89.0)	74.5 (61.0 to 85.0)	76.0 (60.0 to 91.0)	75.0 (62.0 to 88.0)	
Gender, male (%)	50	50	50	50	
*Comorbidities, (yes), n (%)*					
Pulmonary disease, without COPD	1 (10)	1 (10)	0 (0)	1 (10)	0.782
Chronic obstructive pulmonary disease (COPD)	4 (40)	10 (100)	1 (10)	10 (100)	
Hypertension	5 (50)	7 (70)	9 (90)	7 (70)	0.283
Cardiovascular disease	5 (50)	6 (60)	4 (40)	6 (60)	0.776
Diabetic complications			5 (50)	8 (80)	0.350*
Macrovascular disease			5 (50)	7 (70)	0.650*
Microvascular disease			2 (20)	3 (30)	1.000*
Arthritis	3 (30)	7 (70)	3 (30)	2 (20)	0.098
Cancer	3 (30)	2 (20)	1 (10)	2 (20)	0.741
Other diseases	5 (50)	7 (70)	6 (60)	8 (80)	0.532
*CURB-65 score, n (%)*					0.225
0 to 1 (mild)	7 (70)	6 (60)	4 (40)	8 (80)	
2 (moderate)	3 (30)	3 (30)	5 (50)	2 (20)	
** ≥ **3(severe)	0 (0)	1 (10)	1 (10)	0 (0)	
Early warning score, mean (SD)	3.9 (3.3)	4.0 (1.9)	3.5 (2.8)	5.5 (2.0)	0.251
median (range)	4.5 (0 to 9)	4.5 (0 to 6)	3 (0 to 10)	5 (2 to 8)	
*Charlson comorbidity index, n (%)*					–
1 to 2 (mild)	2 (20)	0 (0)	0 (0)	0 (0)	
3 to 4 (moderate)	3 (30)	1 (10)	0 (0)	0 (0)	
≥ 5 (severe)	5 (50)	9 (90)	10 (100)	10 (100)	
*Main laboratory findings*					
HbA1c at admission (mmol/mol), mean (SD)	36.7 (5.0)	37.6 (4.7)	51.9 (9.1)	53.9 (13.3)	–
median (range)	37 (31 to 48)	38 (29 to 43)	50 (42 to 70)	48.5 (45 to 83)	
Antidiabetic medications (yes), n (%)					
Antidiabetics at admission			7 (70)	8 (80)	0.606*
Insulin at admission			4 (40)	4 (40)	1.000*
Non-insulin therapy at admission			5 (50)	6 (60)	0.653*
*IE of insulin per day during hospitalization (IE/day), mean (SD)*	0 (0)	0 (0)	4.7 (6.6)	11.7 (11.6)	0.102*
median (range)	0 (0 to 0)	0 (0 to 0)	1.5 (0.0 to 22.7)	10.1 (0.0 to 43.3)	
*In hospital characteristics*					
Inclusion time in study (days), mean (SD)	4.9 (2.5)	2.3 (0.8)	4.5 (1.5)	3.8 (1.4)	**0.014**
median (range)	4.7 (1.9 to 10.1)	2.3 (1.1 to 3.8)	4.2 (1.9 to 7.0)	3.9 (1.2 to 6.0)	
Length of stay (days), mean (SD)	6.8 (4.0)	4.4 (2.0)	6.0 (2.3)	7.7 (2.9)	0.085
median (range)	5.3 (2.8 to 16.0)	4.7 (2.0 to 8.0)	5.4 (2.0 to 11.4)	8.7 (1.9 to 11.4)	
Need for respiratory support (yes), n (%)	1 (10)	3 (30)	2 (20)	3 (30)	0.665
Need for intensive care (yes), n (%)	0 (0)	0 (0)	0 (0)	1 (10)	0.380
Defect sensor time (hours), mean (SD)	0 (0)	3.06 (8.7)	0 (0)	0 (0)	0.104
median (range)	0 (0 to 0)	0.0 (0 to 24)	0 (0 to 0)	0 (0 to 0)	
Acetaminophen intake (yes), n (%)	10 (100)	6 (60)	6 (60)	1 (10)	**0.001**
*GC before admission (yes), n (%)*	1 (10)	3 (30)	2 (20)	3 (30)	0.665

Bold values denote statistical significance at the *P* ≤ 0.05 level. The range is from minimum to maximum

CAP, community-acquired pneumonia; GC, glucocorticoid intake; T2DM, type 2 diabetes mellitus; IE, International Units

*Comparisons made only for patients with T2DM

The CGM sensors used in this study has an upper detection limit for glucose levels of 22.2 mmol/L (400 mg/dL). Therefore, right-censored glucose values were estimated using a validated imputation model developed previously by authors [19]. A two-sided *P*-value ≤ 0.05 was considered statistically significant for all analyses.

### Setting

This study is part of a large Danish prospective observational cohort study named Surviving Pneumonia conducted at Copenhagen University Hospital of Copenhagen – North Zealand, Denmark. Surviving Pneumonia represents a multipronged initiative to improve and personalize the in-hospital management of patients with CAP.

## Results

### Baseline characteristics (Table 1)

We enrolled 40 patients who were on average 74 (range 55 to 91) years old. Matching was successful among groups (data for food intake not shown). However, inclusion time was lower for group 2 compared to group 1 (P = 0.014), and acetaminophen intake differed from expected values in groups 1 and 4 (both P < 0.01).

### Primary outcomes (Table 2): measurements of GV

**Table 2:** Primary (SD, CV and PPGE) and secondary outcomes (TIR, TAR, TBR, mean glucose level and glycemic gap) for 40 patients hospitalized for community-acquired pneumonia

Parameter	Daytime		Parameter	Nighttime	
	β (95% CI)	*P*		β (95% CI)	*P*
*Relative change in standard deviation (SD) of all CGM-glucose values per change in explanatory variables*					
Intercept	1.16 (0.92 to 1.46)		Intercept	0.71 (0.50 to 1.02)	
T2DM = yes	1.93 (1.40 to 2.66)	** < 0.001**	T2DM = yes	2.29 (1.38 to 3.81)	**0.002**
GC = yes	1.42 (1.04 to 1.97)	**0.031**	GC = yes	1.21 (0.73 to 2.01)	0.451
T2DM·GC = yes	1.09 (0.69 to 1.71)	0.717	T2DM·GC = yes	0.91 (0.44 to 1.86)	0.790

Parameter	Daytime		Parameter	Nighttime	
	β (95% CI)	*P*		β (95% CI)	*P*
*Absolute change in coefficient of variation (CV) in percentage points per change in explanatory variables*					
Intercept	20.3 (16.7 to 23.8)		Intercept	15.0 (10.0 to 20.0)	
T2DM = yes	5.1 (0.0 to 10.1)	**0.046**	T2DM = yes	8.1 (1.0 to 15.2)	**0.026**
GC = yes	0.9 (− 4.1 to 5.9)	0.721	GC = yes	0.5 (− 6.6 to 7.6)	0.887
T2DM·GC = yes	0.9 (− 6.2 to 7.9)	0.805	T2DM·GC = yes	− 3.8 (− 13.8 to 6.2)	0.444

Parameter	Breakfast		Parameter	Lunch		Parameter	Dinner	
	β (95% CI)	*P*		β (95% CI)	P		β (95% CI)	*P*
*Absolute change in post prandial glycemic excursion (PPGE) in mmol/L and mg/dL (at the bottom) per change in explanatory variables*								
Intercept	2.1 (1.3 to 2.9) 38 (23 to 52)		Intercept	1.3 (0.6 to 2.0) 23 (11 to 36)		Intercept	1.3 (0.3 to 2.2) 23 (5 to 40)	
T2DM = yes	1.5 (0.4 to 2.7) 27 (7 to 49)	**0.018**	T2DM = yes	− 0.3 (− 1.3 to 0.7) − 5 (− 23 to 13)	0.602	T2DM = yes	1.4 (− 0.0 to 2.8) 25 (0 to 50)	0.076
GC = yes	0.4 (− 0.9 to 1.7) 7 (− 16 to 31)	0.538	GC = yes	0.3 (− 1.1 to 1.7) 5 (− 20 to 31)	0.671	GC = yes	0.8 (− 0.8 to 2.5) 14 (− 14 to 45)	0.355
T2DM·GC = yes	− 0.3 (− 2.1 to 1.5) − 5 (− 38 to 27)	0.739	T2DM·GC = yes	2.5 (0.7 to 4.3) 45 (13 to 77)	**0.018**	T2DM·GC = yes	− 1.1 (− 3.4 to 1.1) − 20 (− 61 to 20)	0.332

Parameter	Daytime		Parameter	Nighttime	
	β (95% CI)	*P*		β (95% CI)	*P*
*Absolute change in time in range (TIR) in percentage points per change in explanatory variables*					
Intercept	94 (81 to 106)		Intercept	88 (73 to 103)	
T2DM = yes	− 27 (− 44 to − 9)	**0.004**	T2DM = yes	− 2 (− 24 to 19)	0.819
GC = yes	− 11 (− 29 to 6)	0.196	GC = yes	6 (− 15 to 27)	0.554
T2DM·GC = yes	− 31 (− 56 to − 6)	**0.017**	T2DM·GC = yes	− 33 (− 63 to − 3)	**0.032**

Parameter	Daytime		Parameter	Nighttime	
	β (95% CI)	*P*		β (95% CI)	*P*
*Relative change in time above range (TAR) per change in explanatory variables*					
Intercept	2.55 (1.26 to 5.16)		Intercept	0.40 (0.06 to 2.64)	
T2DM = yes	14.19 (6.09 to 33.06)	** < 0.001**	T2DM = yes	42.62 (5.76 to 315.24)	** < 0.001**
GC = yes	7.57 (3.25 to 17.64)	** < 0.001**	GC = yes	38.24 (4.33 to 337.80)	**0.002**
T2DM·GC = yes	0.27 (0.09 to 0.80)	**0.023**	T2DM·GC = yes	0.07 (0.01 to 0.74)	**0.034**

Parameter	Daytime		Parameter	Nighttime	
	β (95% CI)	*P*		β (95% CI)	*P*
*Relative change in time below range (TBR) per change in explanatory variables*					
Intercept	7.39 (3.98 to 13.72)		Intercept		
T2DM = yes	0.16 (0.04 to 0.58)	**0.001**	T2DM = yes	Model is overparametrized	
GC = yes	0.21 (0.06 to 0.78)	**0.026**	GC = yes		
T2DM·GC = yes	2.07 (0.19 to 22.80)	0.556	T2DM·GC = yes		

Parameter	Daytime β (95% CI)			Parameter	Nighttime β (95% CI)		
	mmol/L	mg/dL	*P*		mmol/L	mg/dL	*P*
*Absolute change in mean glucose level in mmol/L and mg/dL per change in explanatory variables*							
Intercept	6.0 (4.4 to 7.5)	108 (79 to 135)		Intercept	5.2 (3.9 to 6.6)	94 (70 to 119)	
T2DM = yes	3.0 (0.8 to 5.2)	54 (14 to 94)	**0.009**	T2DM = yes	2.4 (0.4 to 4.3)	44 (7 to 77)	**0.018**
GC = yes	2.4 (0.1 to 4.6)	43 (2 to 83)	**0.038**	GC = yes	1.6 (− 0.4 to 3.4)	29 (− 7 to 61)	0.113
T2DM·GC = yes	2.5 (− 0.7 to 5.6)	45 (− 13 to 101)	0.120	T2DM·GC = yes	1.2 (− 1.5 to 3.9)	22 (− 27 to 70)	0.374

Parameter	β (95% CI)		*P*
	mmol/L	mg/dL	
*Absolute change in glycemic gap in mmol/L and mg/dL per change in explanatory variables*			
Intercept	2.1 (0.9 to 3.2)	38 (16 to 58)	
T2DM = yes	0.9 (− 0.7 to 2.6)	16 (− 13 to 47)	0.261
GC = yes	1.8 (0.2 to 3.4)	32 (4 to 61)	**0.029**
T2DM·GC = yes	1.3 (− 1.0 to 3.6)	23 (− 18 to 65)	0.259

Bold values denote statistical significance at the* P* ≤ 0.05 level. Each headline for the schemes above  specifies how to interpret the coefficients of each scheme which differs among outcomes due to different statistical approaches

In the following, ( +) and (-) denotes the presence or not, respectively, of T2DM or treatment with GCs.

### Standard deviation (SD) (95%CI)

During the daytime, SD for T2DM + GC- patients increased by a factor of 1.93 (1.40 to 2.66) compared to T2DM- GC- patients (*P* < 0.001). SD for T2DM- GC + patients increased by a factor of 1.42 (1.04 to 1.97) compared to T2DM- GC- patients (*P* = 0.031). During the nighttime, SD for T2DM + GC- patients increased by a factor of 2.29 (1.38 to 3.81) compared to T2DM- GC- patients (*P* < 0.01). GC + status did not affect SD during the nighttime per se (*P* = 0.451). There was no statistically significant interaction between GC treatment and T2DM on SD during the daytime or the nighttime (*P* = 0.717 and *P* = 0.790, respectively).

### Coefficient of variation (CV) (95% CI)

During the daytime, CV of T2DM + GC- patients increased by 5.1 (0.0 to 10.1) percentage points compared to T2DM- GC- patients (*P* = 0.046). During the nighttime, CV for T2DM + GC- patients increased by 8.1 (1.0 to 15.2) percentage points compared to T2DM- GC- patients (*P* = 0.026). CV for T2DM- GC + patients were not statistically different from T2DM- GC- patients during the daytime or the nighttime (*P* = 0.721 and *P* = 0.887, respectively). There was no interaction between T2DM and GC treatment during the daytime or the nighttime on CV (*P* = 0.805 and *P* = 0.444, respectively).

### Postprandial glucose excursions (PPGE) (95% CI)

PPGE increased by 1.5 (0.4 to 2.7) mmol/L (27 (7 to 49) mg/dL) at breakfast for T2DM + GC- patients compared to T2DM- GC- patients (*P* = 0.018). Treatment with GCs was not associated with a significant change in PPGE at all meals. However, at lunch, an interaction between T2DM + and GC + status was found (meaning that the effect of GCs depended on diabetes status) with an increase in postprandial glucose level of 2.5 (0.7 to 4.3) mmol/L (45 (13 to 77) mg/dL) in T2DM + GC + patients compared to T2DM- GC- patients (*P* = 0.018).

### Secondary outcomes (Table 2)

#### Time in range (TIR) (95% CI)

During the daytime, TIR for T2DM + GC- patients was 27 (44 to 9) percentage points lower compared to T2DM- GC- patients (*P* < 0.01) while GC + status did not affect TIR per se. There was an interaction between GC + and T2DM + status with a decrease by 31 (56 to 6) percentage points for T2DM + GC + patients compared to T2DM- GC- patients (*P* = 0.017). During the nighttime, T2DM + and GC + status did not affect TIR per se but an interaction between GC + and T2DM + status was observed (*P* = 0.032).

#### Time above range (TAR) (95%CI)

During the daytime, TAR increased by a factor of 14.19 (6.09 to 33.06) for T2DM + GC- patients and a factor of 7.57 (3.25 to 17.64) for T2DM- CG + patients compared to T2DM- GC- patients (both *P* < 0.001). During the nighttime, TAR for T2DM + GC- and T2DM- GC + patients increased by a factor of 40 compared to T2DM- GC- patients (both *P* < 0.01). Interaction between T2DM + and GC + status for the daytime and the nighttime changed TAR by a factor of 0.27 (0.09 to 0.80) and 0.07 (0.01 to 0.74), respectively, compared to T2DM- GC- patients (*P* = 0.023 and *P* = 0.034, respectively).

#### Time below range (TBR) (95%CI)

During the daytime, TBR was 84 (42 to 96) % lower for T2DM + GC- patients and 79 (22 to 94) % lower for T2DM- GC + patients, compared to T2DM- GC- patients (*P* = 0.001 and *P* = 0.026, respectively). TBR for the nighttime is not reported due to overparameterization of the model.

### Mean glucose level (95%CI)

During the daytime, mean glucose for T2DM + GC- patients increased by 3.0 (0.8 to 5.2) mmol/L (54 (14 to 94) mg/dL) compared to T2DM- GC- patients (*P* < 0.01). Mean glucose increased by 2.4 (0.1 to 4.6) mmol/L (43 (2 to 83) mg/dL) in T2DM- GC + patients compared to T2DM- GC- patients (*P* = 0.038). During the nighttime, mean glucose level were 2.4 (0.4 to 4.3) mmol/L (44 (7 to 77) mg/dL) higher in T2DM + GC- patients compared to T2DM- GC- patients (*P* = 0.018).

### Glycemic gap (95% CI)

The glycemic gap increased by 1.8 (0.2 to 3.4) mmol/L (32 (4 to 61) mg/dL) in T2DM- GC + patients compared to T2DM- GC- patients (*P* = 0.029).

### Post hoc analyses (Table 3)

**Table 3:** Exploratory analyses for primary (SD, CV, PPGE) and secondary (TIR, TAR, TBR, mean glucose level and glycemic gap) outcomes for 40 patients hospitalized for community-acquired pneumonia

Parameter	Daytime		Parameter	Nighttime	
	β (95% CI)	*P*		β (95% CI)	*P*
*Relative change in standard deviation (SD) of all CGM-glucose values per change in explanatory variables*					
Intercept	0.91 (0.42 to 1.96)		Intercept	1.73 (0.54 to 5.55)	
T2DM = yes	1.81 (1.32 to 2.49)	**0.001**	T2DM = yes	2.50 (1.55 to 4.04)	** < 0.001**
GC = yes	1.43 (1.13 to 1.80)	**0.004**	GC = yes	1.18 (0.84 to 1.68)	0.330
HbA1c	1.01 (0.99 to 1.02)	0.121	HbA1c	1.01 (0.98 to 1.03)	0.640
BMI	0.99 (0.97 to 1.02)	0.536	BMI	0.96 (0.93 to 0.99)	**0.012**
CURB-65	1.00 (0.87 to 1.15)	0.987	CURB-65	0.94 (0.76 to 1.15)	0.543

Parameter	Daytime		Parameter	Nighttime	
	β (95% CI)	*P*		β (95% CI)	*P*
*Absolute change in coefficient of variation (CV) in percentage points per change in explanatory variables*					
Intercept	25.8 (13.4 to 38.2)		Intercept	34.4 (18.2 to 50.6)	
T2DM = yes	6.8 (1.7 to 11.9)	**0.010**	T2DM = yes	10.3 (3.6 to 16.9)	**0.004**
GC = yes	1.4 (− 2.3 to 5.1)	0.435	GC = yes	− 0.5 (− 5.4 to 4.3)	0.830
HbA1c	0.0 (− 0.2 to 0.2)	0.938	HbA1c	− 0.1 (− 0.4 to 0.2)	0.607
BMI	− 0.2 (− 0.6 to 0.1)	0.197	BMI	− 0.6 (− 1.1 to − 0.2)	**0.006**
CURB-65	− 0.3 (− 2.5 to 2.0)	0.820	CURB-65	− 0.9 (− 3.8 to 2.0)	0.535

Parameter	Breakfast		Parameter	Lunch		Parameter	Dinner	
	β (95% CI)	*P*		β (95% CI)	*P*		β (95% CI)	*P*
*Absolute change in postprandial glucose excursion (PPGE) in mmol/L and mg/dL (at the bottom) per change in explanatory variables*								
Intercept	4.5 (0.8 to 8.3) 81 (14 to 149)		Intercept	0.3 (− 4.1 to 5.0) 5 (− 74 to 90)		Intercept	− 1.2 (− 5.7 to 3.4) − 22 (− 103 to 61)	
T2DM = yes	1.5 (− 0.1 to 3.2) 27 (− 2 to 58)	0.101	T2DM = 1	− 0.4 (− 2.4 to 1.6) − 7 (− 43 to 29)	0.710	T2DM = yes	− 0.7 (− 2.6 to 1.3) − 13 (− 47 to 23)	0.552
GC = yes	− 1.0 (− 2.2 to 0.2) − 18 (− 40 to 4)	0.151	GC = 1	2.2 (0.8 to 3.7) 40 (14 to 67)	**0.013**	GC = yes	− 0.3 (− 1.7 to 1.2) − 5 (− 31 to 22)	0.730
HbA1c	0.0 (0.0 to 0.1) 0 (0 to 2)	0.722	HbA1c	0.0 (− 0.1 to 0.1) 0 (− 1 to 2)	0.133	HbA1c	0.0 (0.0 to 0.1) 1 (− 1 to 2)	0.291
BMI	− 0.1 (− 0.2 to 0.0) − 2 (− 4 to 0)	0.091	BMI	0.0 (− 0.2 to 0.1) 0 (− 3 to 2)	0.711	BMI	0.0 (− 0.1 to 0.1) 0 (− 2 to 2)	0.868
CURB-65	− 0.1 (− 0.8 to 0.6) − 2 (− 14 to 11)	0.749	CURB − 65	0.8 (− 0.1 to 1.6) 14 (− 2 to 29)	0.133	CURB − 65	1.3 (0.5 to 2.2) 20 (9 to 40)	**0.014**

Parameter	Daytime		Parameter	Nighttime	
	β (95% CI)	*P*		β (95% CI)	*P*
*Absolute change in time in range (TIR) in percentage points per change in explanatory variables*					
Intercept	116.4 (74.6 to 158.1)		Intercept	105.5 (50.7 to 160.2)	
T2DM = yes	− 33.9 (− 51.0 to − 16.7)	** < 0.001**	T2DM = yes	− 12.3 (− 34.8 to 10.2)	0.274
GC = yes	− 23.3 (− 35.8 to − 10.9)	** < 0.001**	GC = yes	− 8.1 (− 24.4 to 8.2)	0.321
HbA1c	− 0.9 (− 1.6 to − 0.2)	**0.018**	HbA1c	− 0.7 (− 1.7 to 0.2)	0.132
BMI	0.5 (− 0.6 to 1.7)	0.372	BMI	0.5 (− 1.0 to 2.0)	0.487
CURB − 65	4.4 (− 3.0 to 11.8)	0.231	CURB-65	4.3 (− 5.4 to 14.0)	0.371

Parameter	Daytime		Parameter	Nighttime	
	β (95% CI)	*P*		β (95% CI)	*P*
*Relative change in time above range (TAR) per change in explanatory variables*					
Intercept	1.06 (0.12 to 9.10)		Intercept	0.38 (0.02 to 9.30)	
T2DM = yes	4.24 (2.12 to 8.48)	** < 0.001**	T2DM = yes	3.26 (0.82 to 12.94)	0.103
GC = yes	3.09 (1.73 to 5.53)	** < 0.001**	GC = yes	3.00 (0.86 to 10.44)	0.095
HbA1c	1.02 (0.99 to 1.06)	0.162	HbA1c	1.03 (0.98 to 1.08)	0.252
BMI	1.02 (0.97 to 1.07)	0.404	BMI	1.04 (0.95 to 1.14)	0.395
CURB-65	1.04 (0.76 to 1.43)	0.795	CURB-65	1.02 (0.48 to 1.17)	0.951

Parameter	Daytime		Parameter	Nighttime	
	β (95% CI)	*P*		β (95% CI)	*P*
*Relative change in time below range (TBR) per change in explanatory variables*					
Intercept	0.01 (0.00 to 69.10)		Intercept	7.43 (0.09 to 602.34)	
T2DM = yes	0.05 (0.01 to 0.37)	**0.006**	T2DM = yes	0.16 (0.03 to 0.89)	**0.044**
GC = yes	0.16 (0.05 to 0.56)	**0.007**	GC = yes	1.63 (0.33 to 7.99)	0.549
HbA1c	1.17 (0.97 to 1.42)	0.103	HbA1c	1.03 (0.94 to 1.13)	0.475
BMI	1.03 (0.90 to 1.18)	0.678	BMI	0.95 (0.84 to 1.07)	0.373
CURB-65	0.92 (0.54 to 1.57)	0.763	CURB-65	1.27 (0.59 to 2.77)	0.546

Parameter	Daytime β (95% CI)			Parameter	Nighttime β (95% CI)		
	mmol/L	mg/dL	*P*		mmol/L	mg/dL	*P*
*Absolute change in mean glucose level in mmol/L per change in explanatory variables*							
Intercept	− 0.7 (− 5.7 to 4.4)	− 13 (− 103 to 79)		Intercept	1.4 (− 3.1 to 5.9)	25 (− 56 to 106)	
T2DM = yes	2.2 (0.1 to 4.2)	40 (2 to 76)	**0.042**	T2DM = yes	1.6 (− 0.2 to 3.5)	29 (− 4 to 63.)	0.084
GC = yes	3.0 (1.5 to 4.5)	− 13 (27 to 81)	** < 0.001**	GC = yes	1.8 (0.5 to 3.1)	32 (9 to 56)	**0.011**
HbA1c	0.1 (0.0 to 0.2)	2 (0 to 4)	**0.003**	HbA1c	0.1 (0.0 to 0.2)	2 (0 to 4)	**0.015**
BMI	0.1 (− 0.1 to 0.2)	2 (− 2 to 4)	0.495	BMI	0.0 (− 0.1 to 0.1)	0 (− 2. to 2)	0.910
CURB-65	− 0.1 (− 0.9 to 0.8)	− 2 (− 16 to 14)	0.904	CURB-65	0.1 (− 0.7 to 0.9)	2 (− 13 to 16)	0.814

Parameter	β (95% CI)		*P*
	mmol/L	mg/dL	
*Absolute change in glycemic gap in mmol/L and mg/dL per change in explanatory variables*			
Intercept	0.1 (− 4.1 to 4.2)	2 (− 74 to 76)	
T2DM = yes	2.0 (0.3 to 3.7)	36 (5 to 67)	**0.021**
GC = yes	2.5 (1.3 to 3.8)	45 (23 to 68)	** < 0.001**
HbA1c	0.0 (− 0.1 to 0.0)	0 (− 2 to 1)	0.370
BMI	0.0 (− 0.1 to 0.1)	0 (− 2 to 2)	0.779
CURB-65	0.0 (− 0.8 to 0.7)	1 (− 14 to 13)	0.954

Model 1 (daytime glycemic variability)			Model 2 (nighttime glycemic variability)		
Parameter	β (95% CI)	P	Parameter	β (95% CI)	P
*Relative change in length of stay (LOS) per change in explanatory variables*					
Intercept	5.85 (2.44 to 14.04)		Intercept	4.09 (2.21 to 7.58)	
T2DM = yes	0.86 (0.52 to 1.41)	0.542	T2DM = yes	0.93 (0.57 to 1.54)	0.780
GC = yes	0.68 (0.43 to 1.07)	0.091	GC = yes	0.80 (0.51 to 1.25)	0.317
Charl. index	1.06 (0.97 to 1.16)	0.185	Charl. index	1.05 (0.96 to 1.16)	0.289
SD daytime	1.28 (1.00 to 1.63)	**0.049**	SD nighttime	1.23 (0.81 to 1.86)	0.327
CV daytime	0.97 (0.45 to 1.01)	0.157	CV nighttime	0.99 (0.95 to 1.03)	0.615

Bold values denote statistical significance at the* P* ≤ 0.05 level. Each headline for the schemes above  specifies how to interpret the coefficients of each scheme which differs among outcomes due to different statistical approaches

BMI, Body Mass Index; Charl. index, Charlson comorbidity index; CURB-65, Community-acquired pneumonia (CAP) severity score; CV, Coefficient of variation; GC, glucocorticoid intake; HbA1c, Haemoglobin A1c; SD, standard deviation of all glucose values; T2DM, type 2 diabetes mellitus

Adding BMI and HbA1c as risk factors for developing GIH and the CURB-65 score to the prespecified models from the primary analysis did not clinically affect the main conclusions from the primary analysis. However, BMI was negatively associated with GV (SD and CV) during the nighttime, and HbA1c was negatively associated with TIR during the daytime, and positively associated with mean glucose level during both the daytime and the nighttime. The CURB-65 score was positively associated with PPGE at dinner.

Adding variables differing at baseline to the analyses did not alter conclusions (data not shown).

### Length of stay (LOS) (95% CI)

LOS increased by 28 (0 to 63) % by every unit increase in SD of all CGM-glucose values during the daytime (*P* = 0.049).

## Discussion

In this study, we found that in-hospital CGM-derived GV (assessed by SD of all CGM-glucose values) in patients with CAP was almost doubled in patients with T2DM not treated with GCs compared to patients without T2DM not treated with GCs. This applies both during the daytime and the nighttime. GC treatment per se increased GV by 42% during the daytime but not during the nighttime. The same pattern did not apply for GV assessed by CV where only a diagnosis of T2DM (but not GCs) increased CV. CV is only weakly correlated with mean glucose compared to SD, which is positively correlated with mean glucose [16]. This might be the reason why GCs had no statistically significant effect on CV. In addition, we found that GC treatment was associated with increased TAR, decreased TBR, increased mean glucose level, and a positive glycemic gap. A high and low glycemic gap has been associated with increased long-term mortality in CAP patients with and without diabetes [20]. Our post hoc analyses revealed that these findings were only slightly confounded by BMI, HbA1c, and CURB-65 score and not affected by variables differing at baseline. This indicates that a diagnosis of diabetes and/or treatment with GCs per se are the most notable markers for the risk of developing glycemic dysregulation in CAP patients. However, there may be other factors such as physical activity that might lower GV, which we did not consider [21]. In addition, COPD in acute exacerbation could per se be related to stress-hyperglycemia [22].

Two other studies with larger populations of 151 [23] and 392 [24] CAP patients (diabetes prevalence 15–20%) receiving GCs showed a higher incidence of hyperglycemia compared to CAP patients not treated with GCs. In these studies, glucose levels were measured by POC blood glucose testing. Our results support these findings. On the contrary, Torres et al. (N = 61) showed no significant effect of GCs on the incidence of hyperglycemia in CAP patients with or without diabetes [25]. This is probably because GCs worsen glycemic outcomes in only a fraction of patients without diabetes, as reviewed by Patel et al. [26]. We found the greatest glycemic dysregulation measured as TIR and PPGE at lunch in CAP patients with T2DM receiving GCs. This indicates an effect modification of T2DM on the effect of GCs on glycemic variables, meaning that the glycemic side-effect of GCs depends on diabetes status. Postprandial hyperglycemia [27], i.e. PPGE, has been correlated with increased long-term mortality in CAP patients, independent of a diabetes diagnosis. Recent studies have demonstrated a relationship between low TIR and the presence of diabetic complications as well as a correlation between low TIR and high HbA1c. Goals for patients with type 2 diabetes (and type 1) is therefore a TIR > 70% [28]. A high TIR can be obtained by decreasing TAR (or decreasing TBR) by treatment with glucose-lowering agents, however, this might increase the risk of hypoglycemia. TIR recommendations for older and high-risk patients with diabetes (e.g. patients with diabetes and hypoglycemic unawareness) should therefore probably be individualized and lowered accordingly [28]. At present, no goal of TIR during hospitalization has been defined.

We found that high HbA1c, irrespective of a diabetes diagnosis, was also a marker for in-hospital glycemic dysregulation. This finding is consistent with previous literature, showing that high HbA1c levels (e.g. patients with diabetes) are linked to an increased risk of developing GIH [5] and that glycemic excursion in patients with diabetes and treated with GCs due to COPD in acute exacerbation are higher than for patients without diabetes measured by CGM [29].

Treatment with GCs lowers both LOS, time to clinical stability, and mortality in CAP patients [6]. However, the development of GIH and increased GV may diminish these beneficial effects [8]. Despite this, treatment of known and new-onset hyperglycemia and a diagnosis of pre-existing diabetes are occasionally ignored in the hospital setting [30]. This is unfortunate since diabetes is associated with an increased risk of pneumonia and COPD [31]. In an international cohort study including 1961 patients with CAP, undiagnosed diabetes was 5%, while the prevalence of prediabetes was 38%. Patients with diabetes might suffer from a decrease in pulmonary function [32] and an approximately threefold higher long-term mortality has been observed in CAP patients with diabetes compared to patients without diabetes [33].

Whether pharmacologically induced normoglycaemia and/or low GV is beneficial in the acute stages of CAP remains controversial [33, 34]. Epidemiological studies have highlighted the negative effects of high GV in CAP patients, focusing especially on an increased LOS [10, 11, 35–37] and increased mortality [10, 27, 36]. Our results support that high GV (measured as SD of all daytime CGM-glucose values) is positively associated with LOS (see Model 1 and Fig. 1). This is worrying considering the ongoing pandemic where GCs are part of the treatment protocol for patients with COVID-19 [38], which could potentially induce great glycemic dysregulation especially in patients with diabetes [19] and prolong LOS and thereby increase work pressure on already overcrowded and stressed wards.

Randomized controlled studies of normoglycemia vs. hyperglycemia on clinical outcomes for patients with CAP are needed to overcome the problems of unrecognized bias in epidemiological studies.

Our findings add to recommendations that screening for GIH should be done 1 to 2 h after the intake of lunch with standard POC capillary blood glucose testing when prednisolone is administered in the morning [39]. This is probably due to the pharmacokinetics of prednisolone, which has the maximal hyperglycemic side-effect 8 h after intake [40]. The recommendations are based on clinical experience and not on prospectively collected data, except for a few newer studies [29, 41].

### Strengths and limitations

This study included a relatively small number of patients (N = 40) distributed on four equal-sized groups, which potentially makes it difficult to find statistically significant differences that may be true (type 2 error). Our many endpoints increase the risk of finding statistically significant differences, by chance, that are not true (type 1 error). It is also a limitation that the CGM-time was only a fraction of the whole hospitalization time (70%), which means that we lost useful glucose information during both the beginning and, to a lesser extent, the end of the patients’ hospitalization. Matching was not successful regarding acetaminophen intake, which potentially can interrupt CGM-glucose readings by falsely increasing glucose levels [42]. We did not take into account that some patients received insulin while others did not. However, the use of corrective insulin was small for patients with diabetes.

We believe that the prospectively collected data on each patient is a strength. Compared to earlier studies, using in-hospital CGM as the primary source of glucose data is a very powerful method since the interval between measurements is only five minutes. With CGM, we were able to quantify the glucose-related variables in CAP patients in a much more accurate way than previously done by standard POC blood glucose testing. Furthermore, the use of blinded CGM ensured objectivity and limited clinical interference during the study period. The precision of CGM during hospitalization has been verified in previous studies [43] albeit technical and practical issues when using in-hospital CGM has also been reported [44].

## Conclusions

In the present study, GV and other glycemic outcomes examined by CGM were studied in a population of hospitalized patients with CAP with or without T2DM and treated or not with GCs. Our results imply that CAP patients with T2DM treated with GCs require great clinical attention due to the increased risk of high GV (especially during the daytime and after lunch) which may limit the recovery from pneumonia. CAP patients without T2DM but treated with GCs were also at risk of developing glycemic dysregulation but to a lesser extent. Our results support that high GV is positively associated with a longer length of stay at the hospital and provide real-world evidence for the clinical experience that a screening procedure for GIH should be done after lunch.

## Supplementary Information

**Additional file 1**. Dataset supporting the findings of this study.

## Data Availability

Data that support the findings of this study are available as Additional file 1.

## Abbreviations

ARDS: Acute respiratory distress syndrome
CAP: Community-acquired pneumonia
CGM: Continuous glucose monitoring
COPD: Chronic obstructive pulmonary disease
CV: Coefficient of variation
GC: Glucocorticoid
GIH: Glucocorticoid-induced hyperglycemia
GV: Glycemic variability
LOS: Length of stay
PPGE: Postprandial glucose excursions
POC: Point of care
SD: Standard deviation
T2DM: Type 2 diabetes mellitus
TAR: Time above range
TBR: Time below range
TIR: Time in range

## References

[1] Ewig S, Birkner N, Strauss R, Schaefer E, Pauletzki J, Bischoff H (2009). New perspectives on community-acquired pneumonia in 388 406 patients. Results from a nationwide mandatory performance measurement programme in healthcare quality. Thorax.

[2] Egelund GB, Jensen AV, Andersen SB, Petersen PT, Lindhardt BØ, von Plessen C (2017). Penicillin treatment for patients with Community-Acquired Pneumonia in Denmark: a retrospective cohort study. BMC Pulm Med.

[3] Kornum J, Thomsen R, Riis A, Lervang H, Schonheyder H, Sorensen H (2007). Type 2 diabetes and pneumonia outcomes. Diabetes Care.

[4] Schuetz P, Friedli N, Grolimund E, Kutz A, Haubitz S, Christ-Crain M (2014). Effect of hyperglycaemia on inflammatory and stress responses and clinical outcome of pneumonia in non-critical-care inpatients: results from an observational cohort study. Diabetologia.

[5] Schultz H, Engelholm SA, Harder E, Pedersen-Bjergaard U, Kristensen PL (2018). Glucocorticoid-induced diabetes in patients with metastatic spinal cord compression. Endocr Connect.

[6] Horita N, Otsuka T, Haranaga S, Namkoong H, Miki M (2015). Adjunctive systemic corticosteroids for hospitalized community-acquired pneumonia: systematic review and meta-analysis 2015 update. Nat Publ Gr.

[7] McAlister FA, Majumdar SR, Blitz S, Rowe BH, Romney J, Marrie TJ (2005). The relation between hyperglycemia and outcomes in 2,471 patients admitted to the hospital with community-acquired pneumonia. Diabetes Care.

[8] Sun B, Luo Z, Zhou J (2021). Comprehensive elaboration of glycemic variability in diabetic macrovascular and microvascular complications. Cardiovasc Diabetol.

[9] Krinsley JS (2008). Glycemic variability: a strong independent predictor of mortality in critically ill patients. Crit Care Med.

[10] Mendez CE, Mok KT, Ata A, Tanenberg RJ, Calles-Escandon J, Umpierrez GE (2013). Increased glycemic variability is independently associated with length of stay and mortality in noncritically ill hospitalized patients. Diabetes Care.

[11] Ferreira L, Moniz AC, Carneiro AS, Miranda AS, Fangueiro C, Fernandes D (2019). The impact of glycemic variability on length of stay and mortality in diabetic patients admitted with community-acquired pneumonia or chronic obstructive pulmonary disease. Diabetes Metab Syndr Clin Res Rev.

[12] Popovic M, Blum CA, Nigro N, Mueller B, Schuetz P, Christ-Crain M (2016). Benefit of adjunct corticosteroids for community-acquired pneumonia in diabetic patients. Diabetologia.

[13] Danne T, Nimri R, Battelino T, Bergenstal RM, Close KL, Devries JH (2017). International consensus on use of continuous glucose monitoring. Diabetes Care.

[14] Nielsen MF, Caumo A, Chandramouli V, Schumann WC, Cobelli C, Landau BR (2004). Impaired basal glucose effectiveness but unaltered fasting glucose release and gluconeogenesis during short-term hypercortisolemia in healthy subjects. Am J Physiol Endocrinol Metab.

[15] Khan R (2001). Postprandial blood glucose. Diabetes Care.

[16] Rodbard D (2018). Glucose variability: a review of clinical applications and research developments. Diabetes Technol Therap.

[17] Diabetes.co.uk - the global diabetes community. Convert HbA1c to Average Blood Sugar Level. 2019. https://www.diabetes.co.uk/hba1c-to-blood-sugar-level-converter.html. Visited between February 2021 and June 2021.

[18] Stasinopoulos DM, Rigby RA (2007). Generalized additive models for location scale and shape (GAMLSS) in R. J Stat Softw.

[19] Klarskov CK, Windum NA, Olsen MT, Dungu AM, Jensen AK, Lindegaard B, Pedersen U, Bjergaard PLK (2021). Telemetric continuous glucose monitoring during the COVID-19 pandemic in isolated hospitalized patients in Denmark—a randomized controlled exploratory trial. Diabetes Technol Ther.

[20] Jensen AV, Baunbæk Egelund G, Bang Andersen S, Petersen PT, Benfield T, Witzenrath M (2019). The glycemic gap and 90-day mortality in community-acquired pneumonia. A prospective cohort study. Ann Am Thorac Soc.

[21] Solomon TPJ, Tarry E, Hudson CO, Fitt AI, Laye MJ (2020). Immediate post-breakfast physical activity improves interstitial postprandial glycemia: a comparison of different activity-meal timings. Pflugers Arch Eur J Physiol.

[22] Dungan KM, Braithwaite SS, Preiser JC (2009). Stress hyperglycaemia. Lancet.

[23] Meijvis SCA, Hardeman H, Remmelts HHF, Heijligenberg R, Rijkers GT, Van Velzen-Blad H (2011). Dexamethasone and length of hospital stay in patients with community-acquired pneumonia: a randomised, double-blind, placebo-controlled trial. Lancet.

[24] Blum CA, Nigro N, Briel M, Schuetz P, Ullmer E, Suter-Widmer I (2015). Adjunct prednisone therapy for patients with community-acquired pneumonia: a multicentre, double-blind, randomised, placebo-controlled trial. Lancet.

[25] Torres A, Sibila O, Ferrer M, Polverino E, Menendez R, Mensa J (2015). Effect of corticosteroids on treatment failure among hospitalized patients with severe community-acquired pneumonia and high inflammatory response: a randomized clinical trial. J Am Med Assoc.

[26] Patel DA, Kristensen PL, Pedersen-bjergaard U, Schultz HH. Glukokortikoidinduceret diabetes og risikofaktorer under højdosisbehandling; 2018.

[27] Koskela HO, Salonen PH, Romppanen J, Niskanen L (2014). Long-term mortality after community-acquired pneumonia—impacts of diabetes and newly discovered hyperglycaemia: a prospective, observational cohort study. BMJ Open.

[28] Battelino T, Danne T, Bergenstal RM, Amiel SA, Beck R, Biester T (2019). Clinical targets for continuous glucose monitoring data interpretation: recommendations from the international consensus on time in range. Diabetes Care.

[29] Burt MG, Roberts GW, Aguilar-Loza NR, Frith P, Stranks SN (2011). Continuous monitoring of circadian glycemic patterns in patients receiving prednisolone for COPD. J Clin Endocrinol Metab.

[30] Kristensen PL, Jessen A, Houe SMM, Banck-Petersen P, Schiøtz C, Hansen KB, Svendsen OL, Almdal TP (2021). Quality of diabetes treatment in four orthopaedic departments in the Capital Region of Denmark. Danish Med J.

[31] Ehrlich SF, Quesenberry CP, Van Den Eeden SK, Shan J, Ferrara A (2010). Patients diagnosed with diabetes are at increased risk for asthma, chronic obstructive pulmonary disease, pulmonary fibrosis, and pneumonia but not lung cancer. Diabetes Care.

[32] Aparna A (2013). Pulmonary function tests in type 2 diabetics and non-diabetic people—a comparative study. J Clin Diagnostic Res.

[33] Jensen AV, Faurholt-Jepsen D, Egelund GB, Andersen SB, Petersen PT, Benfield T (2017). Undiagnosed diabetes mellitus in community-acquired pneumonia: a prospective cohort study. Clin Infect Dis.

[34] Di Yacovo S, Garcia-Vidal C, Viasus D, Adamuz J, Oriol I, Gili F (2013). Clinical features, etiology, and outcomes of community-acquired pneumonia in patients with diabetes mellitus. Med (United States).

[35] Leite SA, Locatelli SB, Niece SP, Oliveira AR, Tockus D, Tosin T (2010). Impact of hyperglycemia on morbidity and mortality, length of hospitalization and rates of re-hospitalization in a general hospital setting in Brazil. Diabetol Metab Syndr.

[36] Akirov A, Diker-Cohen T, Masri-Iraqi H, Shimon I (2017). High glucose variability increases mortality risk in hospitalized patients. J Clin Endocrinol Metab.

[37] Kim Y, Rajan KB, Sims SA, Wroblewski KE, Reutrakul S (2014). Impact of glycemic variability and hypoglycemia on adverse hospital outcomes in non-critically ill patients. Diabetes Res Clin Pract.

[38] Retningslinje til behandling af indlæggelseskrævende voksne patienter med COVID-19. Dansk Selsk Infekt. Published online 2021. https://infmed.dk/covid.

[39] Roberts A, James J, Dhatariya K, Agarwal N, Brake J, Brooks C (2018). Management of hyperglycaemia and steroid (glucocorticoid) therapy: a guideline from the Joint British Diabetes Societies (JBDS) for Inpatient Care group. Diabet Med.

[40] Aberer F, Hochfellner DA, Sourij H, Mader JK (2021). A practical guide for the management of steroid induced hyperglycaemia in the hospital. J Clin Med.

[41] Klarskov CK, Jensen AK, Olsen MT, Windum NA, Pedersen-Bjergaard U, Kristensen PL, et al. The influence of glucocorticoid therapy on glucose metrics in hospitalized patients with diabetes monitored by continuous glucose monitoring n.d.

[42] Maahs DM, Desalvo D, Pyle L, Ly T, Messer L, Clinton P (2015). Effect of acetaminophen on CGM glucose in an outpatient setting. Diabetes Care.

[43] Wang M, Singh LG, Spanakis EK (2019). Advancing the use of CGM devices in a non-ICU setting. J Diabetes Sci Technol.

[44] Klarskov CK, Kristensen PL (2021). Experience from implementing telemetric in-hospital continuous glucose monitoring during the COVID-19 pandemic. J Diabetes Sci Technol.

